# Urinary epidermal growth factor reflects vascular health in boys with either obesity or type 1 diabetes. A role for renin, or beyond?

**DOI:** 10.1371/journal.pone.0283716

**Published:** 2023-03-30

**Authors:** Kristien J. Ledeganck, Annelies Van Eyck, Kristien Wouters, Eline Vermeiren, Benedicte Y. De Winter, Stijn Verhulst, Kim Van Hoorenbeeck, Annick France, Hilde Dotremont, Marieke den Brinker, Dominique Trouet

**Affiliations:** 1 Laboratory of Experimental Medicine and Paediatrics and Member of the Infla-Med Centre of Excellence, University of Antwerp, Antwerp, Belgium; 2 Department of Gastroenterology and Hepatology, Antwerp University Hospital, Edegem, Belgium; 3 Clinical Trial Center, Clinical Research Center Antwerp, Antwerp University Hospital, University of Antwerp, Edegem, Belgium; 4 Department of Paediatrics, Antwerp University Hospital, Edegem, Belgium; 5 Department of Paediatric Endocrinology, Antwerp University Hospital, Antwerp, Belgium; 6 Department of Paediatric Nephrology, Antwerp University Hospital, Antwerp, Belgium; Universidade Federal do Rio de Janeiro, BRAZIL

## Abstract

An increased blood pressure is a known comorbidity of both type 1 diabetes (T1DM) and obesity in children. Increasing evidence suggests a subtle interplay between epidermal growth factor (EGF) and renin along the juxtaglomerular system, regulating the impact of blood pressure on kidney health and the cardiovascular system. In this study, we investigated the relation between urinary EGF, serum renin and blood pressure in children with obesity or T1DM. 147 non-obese children with T1DM and 126 children with obesity, were included. Blood pressure was measured and mean arterial pressure (MAP) and the pulse pressure (PP) were calculated. Serum renin and urinary EGF levels were determined with a commercial ELISA kit. Partial Spearman rank correlation coefficients and multiple linear regression models were used to study the association between renin, the urinary EGF/urinary creatinine ratio and blood pressure parameters. The urinary EGF/urinary creatinine ratio is correlated with the SBP and the MAP in boys with obesity as well as in boys with T1DM. Multiple regression analysis showed that sex and pulse pressure in male subjects were found to be independently associated with renin. Sex, the presence of diabetes, age, the glomerular filtration rate and both pulse pressure and mean arterial pressure in male subjects were independently associated with urinary EGF/urinary creatinine. In conclusion, in boys with either obesity or diabetes, pulse pressure and mean arterial pressure are negatively associated with the functional integrity of the nephron, which is reflected by a decreased expression of urinary EGF.

## Introduction

The burden of childhood obesity is undisputable. According to the World Health Organization, 340 million children and adolescents worldwide were overweight or obese in 2016 [1]. In Belgium, 19% of children and adolescents are overweight and about 5.8% of them are obese [2]. Obesity and its associated comorbidities are related to an increased risk of developing several major health problems, finally resulting in a formerly called ‘metabolic syndrome’ comprising insulin resistance, dyslipidemia, hyperglycemia and hypertension in the context of central obesity. It is known that the components of the metabolic syndrome track from childhood to adulthood and implicate an earlier onset of diabetes and cardiovascular morbidity [3–6].

In parallel with the obesity epidemic, also the incidence of Type 1 diabetes mellitus (T1DM) is increasing in youth [7]. T1DM is an auto-immune metabolic disease with chronic hyperglycaemia. For many patients with T1DM, it is challenging to maintain near-normal glucose blood levels and to reduce the risk of both acute (hypoglycaemia, ketoacidosis) and chronic microvascular (retinopathy, neuropathy and nephropathy) as well as macrovascular complications (hypertension, cardiovascular disease, stroke,…) [8, 9].

Independent of any associated comorbidities, hypertension is a major risk factor for deterioration of the kidney and cardiovascular function in adults [10]. Besides volume and salt overload, the renin-angiotensin system (RAS) plays a major role in the development of renovascular hypertension. Renin is secreted from the juxtaglomerular system in the kidney and elicits a cascade activating angiotensin II (ANG II), which is considered a potent vasoconstrictor of vascular smooth muscle cells as well as a trigger for release of aldosterone from the adrenal cortex [11].

Already in an early phase, tubulo-interstitial changes induced by glomerulopathies lead to diminished production and urinary excretion of epidermal growth factor (EGF), making urinary EGF a sensitive and ‘early stage’ biomarker for the deterioration of kidney function [12]. In children and adolescents with T1DM, it has been shown by our own research group that the urinary EGF is decreased and related to diabetes nephropathy [13]. To the best of our knowledge, no data have been published on urinary EGF in a paediatric population with obesity.

Epidermal growth factor (EGF) stimulates cell growth, proliferation and differentiation by binding to its receptor EGFR [14]. In the kidney, EGF is locally produced in Henle’s loop and in the distal convoluted tubule and is involved in the repairing process of kidney structures [15–17]. On the other hand, excessive stimulation of the EGF receptor has been described to lead to kidney fibrosis and inflammation as well as sodium retention. Moreover, activation of the EGF receptor in tubular cells can be modulated by other ligands such as tumour necrosis factor (TNF) alpha, angiotensin II (Ang II) and high glucose levels [18, 19]. Urinary EGF also plays a role in regulating salt-sensitive hypertension by stimulating urinary salt excretion through epithelial sodium channels (ENaC) in the collecting duct [20]. Finally, the stimulatory effect of (angiotensin-converting enzyme (ACE)-inhibition on urinary EGF excretion [19, 21] provides indirect proof for a suppressive effect of renin on urinary EGF. So increasing evidence suggests an intriguing, subtle interplay between EGF and renin along the juxtaglomerular system, regulating and fine tuning the impact of blood pressure on kidney health and on cardiovascular system.

In this study, we hypothesized that intrarenal renin production plays a role in the fluctuation of urinary EGF excretion, including a suppression of kidney EGF production by high intrarenal renin and vice versa. An increased blood pressure is a known comorbidity in children and adolescents with both T1DM and obesity, however the pathophysiological mechanisms differ between the conditions. In obesity, hypertension is (at least partly) caused by salt-retention and associated with a low to normal renin production [22] which in turn could trigger the kidney EGF production. In contrast, T1DM mediated hypertension is accompanied with hyperfiltration followed by an increase in renin production which hypothetically could depress the tubular EGF production. To investigate these hypotheses, a clinical study was set up in which we included children and adolescents with either T1DM or obesity, and analysed urinary EGF and serum renin in relation to blood pressure parameters.

## Materials and methods

### Study design and patients

#### Paediatric patients with T1DM

Were included at the Antwerp University Hospital (n = 147) between October 2016 and April 2018 in an observational, monocentric, prospective cohort study. Serum and urine samples were collected from each patient.

Inclusion criteria: 8–18 years of age; T1DM diagnosis for at least one year; and insulin treatment of ≥0.5E/kg/d (thereby excluding patients with a significant autonomous insulin production).

Exclusion criteria: obesity defined by a body mass index (BMI) z-score > 2, use diuretics, calcineurin inhibitors, platinum derivates or aminoglycosides and known genetic syndromes.

#### Paediatric patients with obesity and insulin resistance

Were recruited by consecutive inclusion at the paediatric obesity clinic of the Antwerp University Hospital through two research projects (the WELCOME trial, and the ULTROMEC study) (n = 126) [23]. Patients were included between May 2017 and October 2020. Serum and urine samples were collected from each patient. Part of the study population was included via a randomized controlled trial (n◦ISRCTN14722584) of which the results were previously reported [24].

Inclusion criteria: Children with overweight or obesity (aged 8–18 years old) who were diagnosed with insulin resistance. Insulin resistance was defined by meeting one of the following three criteria, depending on a 3-hour oral glucose tolerance test (OGTT): a fasting insulin > 100 pmol/l; or a peak insulin during OGTT > 1000 pmol/l or a glycaemia that exceeds 140 mg/dl at 120’. The OGTT was performed after a 12-hour overnight fast and blood samples were withdrawn at six different moments: 0’, 15’, 30’, 60’, 120’ and 180’. Glucose levels > 140 mg/dl at minute 120 of the OGTT is an indicator of impaired glucose tolerance and therefore of insulin resistance [25, 26].

Exclusion criteria: an underlying illness with intestinal malabsorption, an endogenous cause of obesity (hormonal or genetic), epilepsy, use diuretics, calcineurin inhibitors, platinum derivates or aminoglycosides, T1DM and known genetic syndromes.

In order to avoid overlap between the 2 populations, we explicitly excluded patients with T1DM with a BMI z-score > 2 as well as patients with obesity with characteristics of T1DM.

The study was conducted in accordance with the Declaration of Helsinki and the principles of Good Clinical Practice. The study protocol was reviewed and approved by the Ethics Committee of the Antwerp University Hospital (approval numbers 16/22/2258 (T1DM), 17/10/112 (WELCOME), 19/45/519 (ULTROMEC)). All patients and the parents or legal guardians of the children below 18 years old gave a written informed consent.

### Clinical data

Age, sex, medical and medication history, and laboratory values were extracted out of the electronic patient files for all patients.

#### Anthropometric measurements

Height (cm) was measured to 0.1cm using a standing stadiometer and weight was measured to the nearest 0.05kg using a digital weighing scale.

BMI was calculated as weight in kg over height in m^2^. Weight, height and BMI were further analysed as z-scores using the Flemish Growth Study as reference population [27]. Overweight and obesity were defined according to the International Obesity Task Force (IOTF) criteria [28].

#### Systolic and diastolic blood pressure

The arterial blood pressure was measured three times consecutively by an automated validated oscillometric device at the right upper arm with an adjusted cuff. The mean value of these measurements was calculated. Systolic and diastolic blood pressure z-scores for age, height and sex were calculated [29].

Pulse pressure (PP), a measure for artery stiffness and flow pulsatility [30], (PP) was calculated as the systolic blood pressure (SBP; in mmHg) minus diastolic blood pressure (DBP; in mmHg).

Mean arterial pressure (MAP) was calculated as DBP plus 1/3(PP), and is determined by small resistance artery function and cardiac output [30].

### Laboratory measurements

Serum and urine creatinine were analysed with an automated Dimension Vista 1500 system (Siemens Healthcare Diagnostics, Deerfield, MA, USA).

The estimated glomerular filtration rate (eGFR) was then calculated using the Bedside Swartz equation, which includes serum creatinine and height and is the preferred method in subjects under 18 years old [31].

Urine EGF was measured using an EGF human Elisa kit® (Invitrogen, California, USA), according to the manufacturer’s guidelines. The detection limit of the assay was 3.9 pg/ml. Urine EGF was corrected for kidney function (creatinine) as previously published [32].

Serum renin was measured using a Human Renin Quantikine ELISA kit® (R&D systems, Abingdon, UK), according to the manufacturer’s guidelines [33]. The detection limit of this assay was 31.3 pg/ml.

### Statistical analysis

Normality was tested with Shapiro-Wilk test and visual inspection of QQ-plots. Since not all parameters were normally distributed, patient characteristics were presented per group (obesity/diabetes) as median with range (min-max). As it is known that serum renin levels are higher in boys when compared to age-matched girls [34], all data are shown separately for boys and girls.

The association between renine, the urinary EGF (uEGF)/urinary creatinine (uCreat) ratio and blood parameters was studied in each of the four subgroups (diabetes/obesity by sex) by partial Spearman rank correlation coefficients (rho), controlling for age and height-z-score. Both age and height-z-score were used as controlling variables as children with obesity were seen to be taller than non-obese peers.

Finally, a multiple linear regression model was constructed for renin and log-transformed uEGF/uCreat ratio, containing sex, disease (obesity/diabetes), age, height-z-score, eGFR, PP, MAP and interactions between PP and sex, and between MAP and sex as explanatory variables. In this way a different association was allowed between blood pressure parameters and renin or uEGF/uCreat ratio for boys and girls. Adding an interaction between PP and disease or between MAP and disease did not significantly improve the model fit. Model assumptions were judged by looking at residual plots. Added variable plots were constructed to visualize the relation between renin or uEGF/uCreat ratio and blood pressure parameters after controlling for the remaining variables in the model.

All analyses were performed in R 4.1.3 (R Core Team 2022) and significance was concluded when p-values were below 0.05.

## Results

### Population demographics

#### Group descriptions

As presented in Table 1, 147 normal weighted children and adolescents with T1DM and 126 children and adolescents with obesity, were included into this study.

**Table 1:** Characteristics of patients with obesity and T1DM.

	Obesity			T1DM		
	All	Boys	Girls	All	Boys	Girls
**General**						
**N**	126	57	69	147	83	64
Age (y)	12.8 (8.1–17.4)	12.0 (8.92–16.7)	13.2 (8.11–17.4)	13.5 (3.70–17.9)	13.6 (5.68–17.8)	13.4 (3.70–17.9)
Height (cm)	161 (131–187)	158 (135–187)	162 (131–178)	160 (101–195)	163 (115–194)	158 (101–182)
Height z-score	1.18 (-1.07–4.06)	1.19 (-1.07–3.92)	1.07 (-0.76–4.06)	-0.06 (-3.17–2.70)	0.01 (-2.51–2.70)	-0.18 (-3.17–2.65)
BMI (kg/m²)	31.5 (18.2–48.7)	31.0 (22.1–39.1)	31.6 (18.2–48.7)	19.2 (12.0–28.9)	18.9 (14.0–26.9)	19.9 (12.0–28.9)
BMI z-score	2.49 (1.89–3.30)	2.41 (1.90–3.20)	2.50 (1.89–3.30)	0.34 (-4.28–1.83)	0.26 (-1.83–1.70)	0.40 (-4.28–1.83)
**Blood pressure**						
N	126	57	69	139	78	61
SBP (mmHg)	113 (87–170)	115 (87–148)	111 (87–170)	117 (89–154)	118 (93–154)	116 (89–140)
DBP (mmHg)	68 (46–106)	68 (46–92)	68 (48–106)	66 (41–90)	65 (47–89)	68 (41–90)
SBP z-score^a^	0.66 (-1.88–2.33)	0.81 (-1.64–2.33)	0.41 (-1.88–2.33)	0.88 (-1.55–2.33)	0.74 (-1.23–2.33)	1.17 (-1.55–2.33)
DBP z-score^a^	0.37 (-1.75–2.33)	0.50 (-1.34–2.33)	0.31 (-1.75–2.33)	0.33 (-1.75–2.33)	0.14 (-1.28–2.33)	0.52 (-1.75–2.33)
MAP	82 (62–119)	83 (64–103)	82 (62–119)	83 (61–101)	83 (67–101)	84 (61–101)
PP	45 (20–76)	47 (21–71)	44 (20–76)	48 (25–85)	51 (27–85)	46 (25–68)
**Nephrological parameters**						
N	125	56	69	147	83	64
eGFR (ml/min/m²)	120.1 (77.3–175.2)	120.8 (81.7–175.2)	120.1 (77.3–173.1)	109.4 (78.7–168.4)	110.1 (82.4–168.4)	106.7 (78.7–158.9)
N	108	49	59	146	83	63
Renin (pg/ml)	701.5 (265.8–1728.2)	770.7 (437.6–1689.4)	677.5 (265.8–1728.2)	763.5 (224.0–2672.0)	880.8 (425.9–2672.0)	661.2 (224.0–2156.0)
N	97	43	54	103	64	39
uEGF/uCreat (ng/mg)	61.4 (12.3–183.4)	67.3 (12.3–183.4)	54.6 (27.1–106.4)	33.09 (6.3–96.4)	32.7 (6.3–96.4)	34.8 (15.5–94.4)

Table 1: Descriptive data for children and adolescents with obesity (left panel) and T1DM (right panel). Data are presented as median (range) for both patient groups with subsequently boys and girls separately for each group. BMI: body mass index; SBP: systolic blood pressure; DBP: diastolic blood pressure; MAP: mean arterial pressure; PP: pulse pressure; eGFR: estimated glomerular filtration rate; uEGF: urinary EGF; uCreat: urinary creatinine. ^a^ SBP-z-score and DBP-z-score missing for 2 boys with obesity because of height-z > 3

Based on the height z-scores, we see that children and adolescents with obesity were taller than their peers and had an increased BMI z-score meeting the criteria for obesity as described by the IOTF. The z-scores for height and BMI in children and adolescents with T1DM were within normal range.

Both in boys with T1DM and boys with obesity, the serum renin levels are higher when compared to girls.

**Fig 1:**
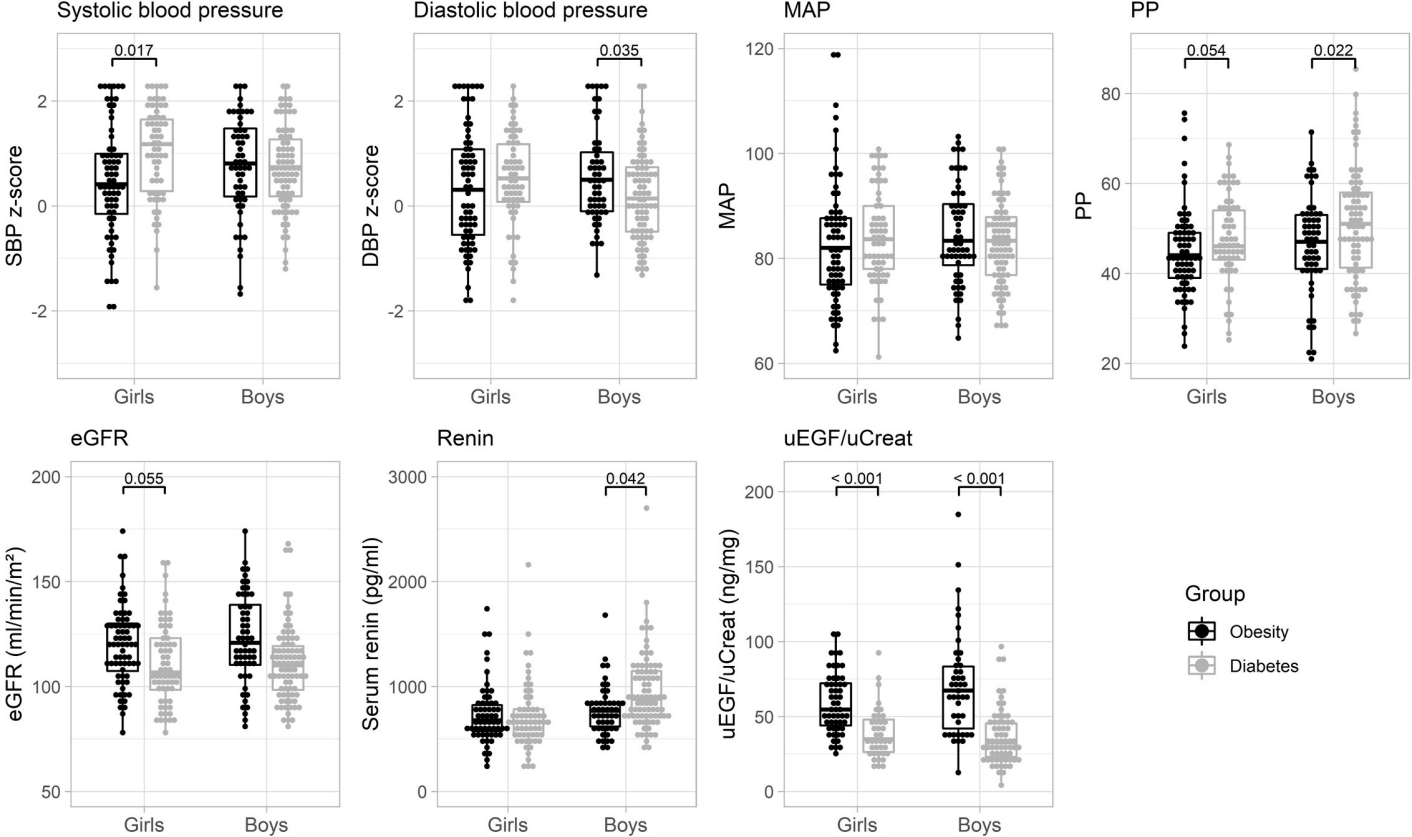
Boxplots represent differences in blood pressure related variables (upper panel) and kidney related variables (lower panel) between boys and girls with T1DM (light grey) and obesity (black). MAP: mean arterial pressure; PP: pulse pressure; eGFR: estimated glomerular filtration rate; uEGF: urinary epidermal growth factor; uCreat: urinary creatinine.

Pulse pressure was significantly higher in the T1DM group compared to the group with obesity (p < 0.001) after correction for age, sex and height-z-score, while no significant difference in MAP could be withheld. Overall, no significant difference in SBP z-score or DBP z-score was seen between the subpopulations with obesity or diabetes. Five percent (7/139) of the children and adolescents with diabetes had a SBP of more than 5 mmHg above 95^th^ centile and 2.2% (3/139) displayed a DBP of more than 5 mmHg above 95^th^ centile (called pre-hypertension or stage 1 hypertension). In the subgroup with obesity, 7.3% (9/124) of the children and adolescents were in the pre-hypertensive range for SBP and 8.1% (10/124) for DBP. The criteria for stage 2 hypertension (> 5 mmHg above P99 for age and height) were only met in 2.2% of the T1DM group (0.7% DBP (1/139) and 1.4% SBP (2/139)), and in 4.0% of the population with obesity (2.4% DBP (3/124) and 2.4% SBP (3/124)). The boxplots shown in Fig 1, represent differences between boys and girls with T1DM and obesity for blood pressure related variables and kidney related variables.

In children and adolescents with T1DM, proteinuria was 14.5 (10.0–20.8) mg/dl and in children and adolescents with obesity 12.6 (8.2–18.0) mg/dl (p = 0.016). In children and adolescents with T1DM, microalbuminuria was 8.4 (5.6–12.6) mg/g creatinine and in children and adolescents with obesity 5.5 (2.9–7.8) mg/g creatinine (p<0.001). Eight percent of the children and adolescents with obesity and 10% of the children and adolescents with T1DM had microalbuminuria >30mg/g creatinine (p = 0.630).

### Partial correlation analysis

Partial correlations controlling for age and height z-score between renin, urinary EGF/ urinary creatinine ratio, blood pressure parameters and eGFR are depicted in Table 2. Both in boys with obesity as well as boys with T1DM the urinary EGF/urinary creatinine ratio is significantly correlated with the SBP and the MAP. No significant correlations were withheld in girls. The serum renin levels were not correlated with the urinary EGF/urinary creatinine ratio, the glomerular filtration rate nor blood pressure.

**Table 2:** Partial correlations, controlling for age and height z-score, between renin, uEGF/uCreatinine eGFR and blood pressure parameters in boys and girls with obesity or T1DM.

	Boys			Girls		
**Obesity**		Renin	uEGF/uCreat		Renin	uEGF/uCreat
	uEGF/uCreat	ρ = -0.067		uEGF/uCreat	ρ = 0.22	
		(p = 0.69)			(p = 0.15)	
	eGFR	ρ = -0.22	ρ = 0.093	eGFR	ρ = -0.11	ρ = 0.22
		(p = 0.14)	(p = 0.56)		(p = 0.40)	(p = 0.11)
	SBP z-score	ρ = -0.04	**ρ = -0.38**	SBP z-score	ρ = -0.053	ρ = 0.11
		(p = 0.79)	**(p = 0.016)**		(p = 0.69)	(p = 0.46)
	DBP z-score	ρ = -0.23	ρ = -0.30	DBP z-score	ρ = 0.056	ρ = 0.27
		(p = 0.12)	(p = 0.062)		(p = 0.68)	(p = 0.056)
	MAP	ρ = -0.066	**ρ = -0.33**	MAP	ρ = 0.016	ρ = 0.24
		(p = 0.66)	**(p = 0.037)**		(p = 0.90)	(p = 0.089)
	PP	ρ = 0.095	ρ = -0.18	PP	ρ = -0.18	ρ = -0.026
		(p = 0.53)	(p = 0.26)		(p = 0.17)	(p = 0.86)
**Diabetes**		Renin	uEGF/uCreat		Renin	uEGF/uCreat
	uEGF/uCreat	ρ = -0.11		uEGF/uCreat	ρ = -0.099	
		(p = 0.40)			(p = 0.56)	
	eGFR	ρ = 0.18	ρ = 0.22	eGFR	ρ = 0.23	ρ = 0.15
		(p = 0.11)	(p = 0.092)		(p = 0.07)	(p = 0.36)
	SBPz-score	ρ = 0.039	**ρ = -0.27**	SBPz-score	ρ = 0.058	ρ = 0.20
		(p = 0.74)	**(p = 0.041)**		(p = 0.67)	(p = 0.25)
	DBPz-score	ρ = 0.030	ρ = -0.25	DBPz-score	ρ = -0.078	ρ = 0.054
		(p = 0.79)	(p = 0.059)		(p = 0.56)	(p = 0.76)
	MAP	ρ = 0.063	**ρ = -0.38**	MAP	ρ = -0.002	ρ = 0.041
		(p = 0.59)	**(p = 0.007)**		(p = 0.99)	(p = 0.81)
	PP	ρ = 0.019	ρ = -0.18	PP	ρ = 0.11	ρ = 0.14
		(p = 0.87)	(p = 0.17)		(p = 0.41)	(p = 0.43)

Table 2: In this table the partial correlations are presented between renin and related parameters and uEGF/uCreatinine and related parameters in boys (left panels) and girls (right panels) with obesity (upper panels) or T1DM (lower panels). Spearman’s rho (ρ) controlled for age and height-z-score are shown and the respective p-values. Significant relations are displayed bold (p<0.05). SBP: systolic blood pressure; DBP: diastolic blood pressure; MAP: mean arterial pressure; PP: pulse pressure.

### Regression analysis

**Fig 2:**
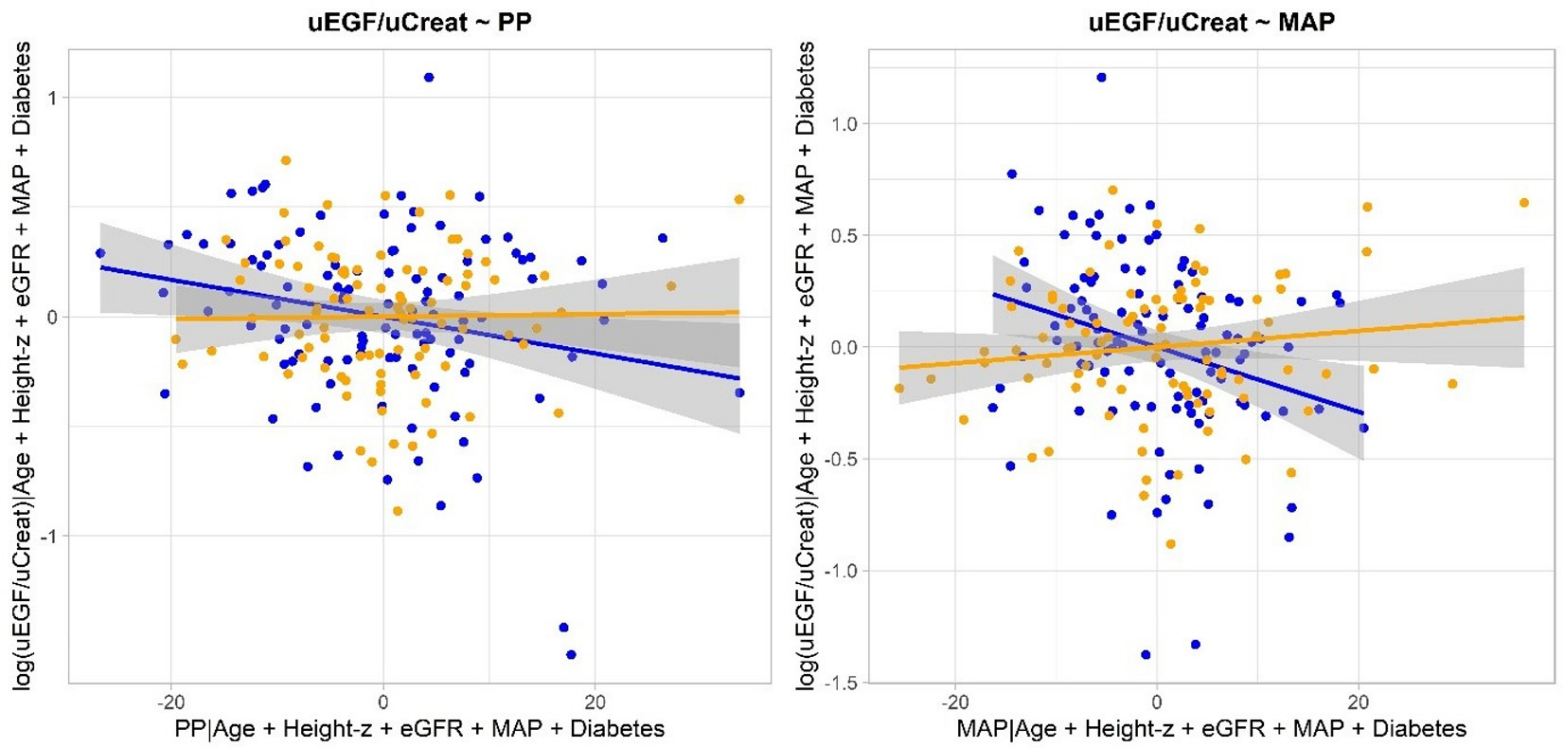
The added variable plots visualize the relation between uEGF/uCreat and respectively PP and MAP, after correction for the other covariates in the linear regression model (i.e. age, height z-score, eGFR and diabetes). The darker (blue) dots and lines correspond with the boys, while the lighter (orange) dots and lines depict the girls. As seen in the model, no clear relationship is seen for the girls, while in the boys we see a negative association between uEGF/uCreat and PP or MAP. MAP: mean arterial pressure; PP: pulse pressure; EGF: epidermal growth factor.

Two multiple linear regressions models were fitted to determine which parameters are independently related to renin or urinary EGF/urinary creatinine as presented in Table 3. Sex and the pulse pressure in male subjects were found to be independently associated with renin while disease (diabetes or obesity), age, height z-score, glomerular filtration and MAP were not. Sex, the presence of diabetes, age, the glomerular filtration rate and both pulse pressure and mean arterial pressure in male subjects appeared to be independently associated with urinary EGF/urinary creatinine. The added variable plots in Fig 2 visualize the relation between uEGF/uCreat and respectively PP and MAP, after correction for the other covariates in the linear regression model (i.e. age, height-z-score, eGFR and diabetes). The darker (blue) dots and lines correspond with the boys, while the lighter (orange) dots and lines depict the girls. As seen in the model, no clear relationship is seen for the girls, while in the boys we see a negative association between uEGF/uCreat and PP or MAP.

**Table 3:** Multiple linear regression models.

	Renin		Urinary EGF/Creatinine (log)	
	Coeff (SE)	P-value	Coeff (SE)	P-value
Sex: Male (ref female)	-860.42 (415.27)	**0.039**	1.98 (0.51)	**<0.001**
Disease: Diabetes (ref obesity)	68.62 (47.84)	0.15	-0.50 (0.062)	**< 0.001**
Age	5.18 (9.25)	0.58	-0.067 (0.012)	**< 0.001**
Height-z-score	-23.30 (20.29)	0.25	-0.022 (0.026)	0.40
eGFR	1.13 (1.22)	0.36	0.005 (0.002)	**0.005**
PP–Female	-4.51 (3.09)	0.15	0.001 (0.004)	0.78
PP–Male	6.12 (2.46)	**0.014**	-0.009 (0.003)	**0.006**
MAP–Female	-3.58 (2.85)	0.21	0.004 (0.003)	0.28
MAP–Male	2.49 (3.44)	0.47	-0.014 (0.004)	**0.001**

Table 3: Multiple linear regression models for renin (left panel) and urinary EGF/creatinine (right panel). Significant relations are displayed bold (p<0.05). SBP: systolic blood pressure; DBP: diastolic blood pressure; MAP: mean arterial pressure; PP: pulse pressure.

## Discussion

Aiming at dissecting a hypothetical interaction between systemic blood pressure, plasma renin and urinary EGF in children, we compared the characteristics of these three actors in two distinct paediatric populations, namely children with obesity and T1DM, thereby avoiding any overlap of disease. Although no direct link between renin and urinary EGF could be withheld, two interesting patterns of association were observed in male subjects: pulse pressure was found to be independently related to renin, and both pulse pressure and mean arterial pressure showed a clear association with urinary EGF.

### Systemic blood pressure

Vascular health is determined by arterial vascular stiffness, which in turn comprises both large artery elasticity (non-invasively measured by pulse pressure (PP)) and small artery compliance (indirectly represented by mean arterial pressure (MAP)) [30]. Systemic blood pressure reflects the functional condition of the arterial vessel wall. Already at childhood age, a limitation of large artery elasticity can be detected by pulse wave velocity (correlating with pulse pressure) before vascular wall thickening becomes apparent [35, 36]. In children, and particularly adolescents with either T1DM or obesity, systolic blood pressure has a major contribution to both increased stiffness of large arteries and structural vascular wall thickening [35–37]. Therefore, four components of systemic blood pressure, namely systolic BP, diastolic BP, PP and MAP, should be taken into account when evaluating vascular health in a study population. Mapping these 4 blood pressure components in the comparison of our 2 study groups, we observed that pulse pressure was significantly higher in the T1DM group compared to the obese (p < 0.001) after correction for age, sex and height z-score, while no significant difference in MAP could be withheld. Overall, no significant differences in SBP z-score or DBP z-score were seen between the subpopulations with obesity or diabetes. According to the epidemiologic criteria of hypertension in children [38], thus taking age and height into account, 5.0% (7/139) of the children with diabetes had a SBP of more than 5 mmHg above 95^th^ centile and 2.2% (3/139) displayed a DBP of more than 5 mmHg above 95^th^ centile (called pre-hypertension or stage 1 hypertension). In the subgroup with obesity, 7.3% (9/124) of the children were in the pre-hypertensive range for SBP and 8.1% (10/124) for DBP. The criteria for stage 2 hypertension (> 5 mmHg above P99 for age and height) were only met in 2.2% of the T1DM group (0.7% DBP (1/139) and 1.4% SBP (2/139)), and in 4.0% of the population with obesity (2.4% DBP (3/124) and 2.4% SBP (3/124)).

### Plasma renin

Children with obesity had lower renin levels compared to the paediatric T1DM group although this difference was only significant in the male subjects. The lower renin levels observed in the subjects with obesity correspond to the well-documented difference in pathophysiological cause of hypertension between obese and diabetic subjects, namely salt-driven low or normal renin hypertension in obesity [22] opposed to renin-induced renovascular hypertension in T1DM patients. When comparing subgroups by sex, this significance could only be confirmed in boys (p = 0.042), which is in line with the recently described differences in renin levels according to sex during puberty [34]. From the age of 12 years on, sex-hormones appear to have a major influence on the blood level of renin, being more pronounced in males [34]. Moreover, in girls, it has recently been shown that pubertal development has a major effect on blood pressure [39]. We presume that the complex endocrine influence on blood renin as well as on blood pressure during adolescence might partly explain why no associations could be withheld in the partial correlation analyses for renin with any of the blood pressure parameters. However, when taking urinary EGF/creatinine into account in the multiple linear regression model focusing on renin, both pulse pressure and sex appeared to be independently associated with renin. We will elaborate on this finding in the section on pulse pressure, plasma renin and urinary EGF of the discussion (see below).

### Urinary EGF

The third player in our study was urinary EGF/creatinine, which showed to be significantly lower in de T1DM group compared to the patients with obesity (p < 0.001 after correction for age, height z-score and sex). The patients with diabetes had a lower, although not significant (p = 0.10) eGFR than the children with obesity. However, in the partial correlation analyses, no significant link between urinary EGF/creatinine and eGFR was withheld in any of the subgroups, indicating that the decreased urinary excretion of EGF in the diabetic subgroup was not merely a reflection of a lower eGFR. As published before, a decreasing urinary EGF might precede the decline in glomerular filtration rate in patients with T1DM [13].

Strikingly, in boys with obesity as well as in boys with diabetes, the partial correlation analysis did reveal a significant, inverse link between urinary EGF/creatinine and both SBP and MAP. The inverse correlation between urinary EGF/creatinine and MAP in the male patients was confirmed in the multiple linear regression model, and appeared to be independent of disease (T1DM or obesity) and independent of eGFR. It is known that, besides its function as sensitive, pre-clinical biomarker for kidney function and tubulo-interstitial fibrosis, urinary EGF plays a role in regulating salt-sensitive hypertension. In hypertensive adults, salt-infusion resulted in an increased urinary expression of EGF, which in turn triggered urinary salt excretion through ENaC channels in the collecting duct [20]. So a direct tubular effect of increased ‘salt-sensitive’ blood pressure on urinary EGF would rather result in a raised EGF excretion. Although our study patients were not overt hypertensive, the inverse association of blood pressure and urinary EGF/creatinine therefore seems to exclude a similar direct tubular response of urinary EGF activation on salt overload in our population. Our data rather suggest a chronic state of hemodynamic overload in the glomerular microcirculation, where the decrease in urinary EGF could be interpreted as an early sign of beginning latent glomerular changes (podocyte detachment) preceding initial signs of kidney damage.

On the other hand, the significant difference between urinary EGF/creatinine levels in the children with obesity or diabetes, seems to confirm the difference in physiological regulation of blood pressure in the 2 groups, salt-sensitivity-driven in the obese versus renin-induced in the T1DM patients.

### Plasma renin and urinary EGF

Our study could not provide evidence for a direct link between renin and urinary EGF/creatinine. Of course, we should keep in mind that the measured serum renin is only a weak surrogate of the actual intrarenal RAS, and thus possibly not strong or reliable enough to uncover intrarenal actions between RAS and urinary EGF.

Reviewing the literature, no direct effect of renin on urinary EGF has been demonstrated so far. In a few reports, the influence of the intrarenal RAS on urinary EGF has been indirectly studied by the effect of ACE-inhibition (ACE-I): in a rat model inducing RAS activation (by salt-depletion), a beneficial stimulatory effect of ACE-I on urinary EGF has been observed [19]. In humans, a raise of urinary EGF after treatment with ACE-I in hypertensive adult patients with diabetes similarly suggests that activation of RAS inhibits urinary EGF excretion [21]. Our data in children and adolescents are in line with the indirect evidence obtained in these studies: multiple linear regression models applied in our study show a positive link between renin and pulse pressure, as well as an inverse correlation between pulse pressure and urinary EGF/creatinine in male subjects.

### Pulse pressure, plasma renin and urinary EGF

Pulse pressure is the pulsatile component of the blood pressure, and reflects large arterial stiffness [30]. A gradual decline in kidney function with higher pulse pressure is observed in healthy adults, and this effect is more pronounced in the presence of diabetes or obesity [10]. Particularly in diabetic adult patients, an increase in pulse pressure is associated with proteinuria and micro-albuminuria, despite a normal range systolic blood pressure [40]. Although a raised pulse pressure has been described both in children and adolescents with diabetes [41] or obesity [42], the effect of increased pulse pressure on glomerular integrity, which appears detrimental at adult age, has not yet been studied in children.

The positive correlation between pulse pressure and plasma renin which we observed in the subpopulation of boys with either obesity or T1DM, does not provide an answer on which of these 2 actors triggers the other. A higher pulse pressure could theoretically be a consequence of RAS activation, but the lack of statistical evidence in the partial correlations (Table 2) for any association between renin and MAP or SBP in the separate subgroups (obesity and diabetes) does not favour a primary role for renin in the cascade, and rather suggests the opposite: pulse pressure induces early hemodynamic alterations in the glomerular microcirculation resulting in intrarenal RAS activation, prior to any other measurable sign of glomerular damage such as micro-albuminuria, proteinuria or decline in kidney function. The observation in the multiple linear regression models that pulse pressure is inversely associated with urinary EGF (which has been shown to be a very sensitive biomarker for early kidney deterioration), reinforces the idea that pulse pressure leads to early kidney deterioration by offending glomerular integrity through increased hemodynamic glomerular disturbance.

### Mean arterial pressure and urinary EGF

Complementary to pulse pressure, mean arterial pressure (MAP) represents the steady reflective component of blood pressure, and is determined by cardiac output and systemic vascular resistance of the small arteries [30]. In order to maintain appropriate perfusion of vital organs, MAP is tightly regulated through a complex interaction between cardiovascular, kidney and autonomic nervous systems. The kidney system affects MAP directly via RAS, and helps to maintain MAP through regulation of plasma volume, thereby affecting cardiac output. In healthy, non-hypertensive adults, a higher MAP has been significantly linked to glomerular injury by podocyte detachment, and not to any marker of direct tubular cell injury [43]. Consecutive podocyte loss from the glomerulus into the urine results in progression of glomerular damage and finally glomerulosclerosis [44].

In our study population, a significant inverse correlation between MAP and urinary EGF was observed in the subgroup of boys with either T1DM or obesity, independent of eGFR. So again, even before any obvious parameter of kidney decline (such as proteinuria or decrease in GFR) becomes apparent, a lower urinary EGF in our population seems to reflect the adverse effect of systemic blood pressure on glomerular integrity, preceding effective kidney decline.

To the best of our knowledge, the observations in our study are the first to provide insight in the urinary excretion of EGF in children with obesity. Moreover, the association between PP and MAP on the one hand and the excretion of urinary EGF in boys with either obesity or T1DM on the other hand, also represents a novel finding that has not been reported before, not even in adults. So additionally to the known function of urinary EGF as an early prognostic biomarker for kidney damage, urinary EGF can also be considered as a sensitive alarm bell for preclinical undermined vascular health in obesity and T1DM at young age, long before the development of overt hypertension.

Although no direct effect of renin on urinary EGF excretion could be demonstrated in this study, extrapolation of the results provides arguments for an either triggering and/or amplifying role of renin in the observed negative loop between blood pressure and urinary EGF in this pediatric population.

In the prevention of vascular disease in children with diabetes or obesity, we could even argue that, besides physical exercise and dietary measurement, a lower threshold for ‘preventive’ ACE-inhibition should be envisaged in order to break the vicious circle between increased blood pressure and early signs of impaired vascular health and of glomerular damage. The current decisive algorithm of when to start ACE-inhibition and / or diuretics in children with diabetes or obesity [45] relies on systolic and diastolic blood pressure and on renin blood levels. We believe to have enough arguments to promote urinary EGF as a very sensitive barometer of vascular condition in children with obesity or diabetes, which should be taken into account in such a preventive algorithm.

Our findings also shed a new light on the definition of hypertension in childhood, which currently is still based on the upper segment of normal BP distribution [29], and not on long-term outcome. The decreased excretion of urinary EGF at young age which correlates with higher pulse pressure, illustrates that the percentiles nowadays used to define (pre-)hypertension in children and adolescents underestimate the longitudinal risk, and provides arguments for a lower level to define high risk BP.

### Strengths and limitations

Strengths: By including two well-defined populations of lean children and adolescents with T1DM and children and adolescents with obesity and insulin resistance, we were able to investigate the relationship between renin, EGF and vascular status in two important patient groups of the metabolic spectrum. The young age implies a purer study population (compared to the adult population) with hardly any co-morbidity or bias from confounding factors of metabolic syndrome (obesity) or end organ damage (diabetes). The children and adolescents who participated in this study were not treated for hypertension. This rules out the possibility that drug treatment influenced the results. Moreover, they had a normal eGFR. Both blood pressure and renin production can be impaired in kidney failure [46], so if kidney function was impaired, this could influence the results, which is not the case in our study population.

Limitations: The study is limited by the fact that serum renin was used as a surrogate for intrarenal RAS. Both plasma and serum renin have been analysed in other studies as measures of renal RAS [22, 34, 46, 47], although it remains a proxy for actual intrarenal renin production. Although there are many advantages of investigating a pediatric population and providing new insights (e.g. time of onset of disturbed physiological processes and the absence of comorbidities), the hormonal fluctuations that occur during puberty by fluctuations in testosterone and estrogens levels may affect renin levels. The pubertal stages were not determined in this study, so unfortunately, we were not able to correct for this. The children with T1DM were slightly older than those with obesity, so differences between these two groups are possibly due to hormonal influences during puberty. Although blood pressure z-scores, PP and MAP are representative for arterial hypertension, arterial stiffness and small resistance artery function and cardiac output respectively, other measures like echocardiographic parameters or pulse wave velocity could provide more detailed information on the hypertensive condition and endothelial function.

Future perspectives: Future studies should focus on evaluating the individual evolution of urinary EGF, renin and blood pressure under the influence of weight loss in children and adolescents with obesity, or the effect of physical exercise in both children and adolescents with obesity or T1DM. It would be interesting to implement a roadmap in future studies in which the measurement of a mean blood pressure over 24h and overnight blood pressure measurement can be decided based on decreased urinary EGF levels. This might highlight the function of urinary EGF in selecting patients with nightly dipping and thus at risk of early cardiovascular morbidity.

## Conclusion

In boys with either obesity or diabetes, pulse pressure and mean arterial pressure are negatively associated with the functional integrity of the nephron (altered hemodynamic glomerular microcirculation, disturbing intrarenal RAS production), which is reflected by a decreased expression of urinary EGF. So in the prevention of cardiovascular morbidity in children with either obesity or diabetes, urinary EGF measurement could play an important role to monitor the earliest phase of impaired vascular health and of preclinical kidney damage.

## References

[1] World Health Organization. Obesity and overweight 2016. Available from: http://www.who.int/mediacentre/factsheets/fs311/en/.

[2] Drieskens S, Charafeddine R, Gisle L. Gezondheidsenquête 2018: Voedinsstatus. Brussel, Belgium: Sciensano; 2018 D/2019/14.440/53.

[3] Nguyen QM, Srinivasan SR, Xu JH, Chen W, Berenson GS. Changes in risk variables of metabolic syndrome since childhood in pre-diabetic and type 2 diabetic subjects: the Bogalusa Heart Study. Diabetes care. 2008;31(10):2044–9. Epub 2008/07/17. doi: 10.2337/dc08-0898 ; PubMed Central PMCID: PMC2551652. 18628566 PMC2551652

[4] Garnett SP, Baur LA, Srinivasan S, Lee JW, Cowell CT. Body mass index and waist circumference in midchildhood and adverse cardiovascular disease risk clustering in adolescence. Am J Clin Nutr. 2007;86(3):549–55. Epub 2007/09/08. PubMed doi: 10.1093/ajcn/86.3.549 . 17823416

[5] Chen W, Srinivasan SR, Li S, Xu J, Berenson GS. Clustering of long-term trends in metabolic syndrome variables from childhood to adulthood in Blacks and Whites: the Bogalusa Heart Study. American journal of epidemiology. 2007;166(5):527–33. Epub 2007/06/19. doi: 10.1093/aje/kwm105 . 17573336

[6] Srinivasan SR, Frontini MG, Berenson GS. Longitudinal changes in risk variables of insulin resistance syndrome from childhood to young adulthood in offspring of parents with type 2 diabetes: the Bogalusa Heart Study. Metabolism: clinical and experimental. 2003;52(4):443–50; discussion 51–3. Epub 2003/04/18. doi: 10.1053/meta.2003.50065 . 12701056

[7] March CA, Becker DJ, Libman IM. Nutrition and Obesity in the Pathogenesis of Youth-Onset Type 1 Diabetes and Its Complications. Front Endocrinol (Lausanne). 2021;12:622901. Epub 2021/04/09. doi: 10.3389/fendo.2021.622901 ; PubMed Central PMCID: PMC8021094. 33828529 PMC8021094

[8] Bergen K, Mobarrez F, Jorneskog G, Wallen H, Tehrani S. Phosphatidylserine expressing microvesicles in relation to microvascular complications in type 1 diabetes. Thromb Res. 2018;172:158–64. Epub 2018/11/18. doi: 10.1016/j.thromres.2018.10.026 . 30447538

[9] DiMeglio LA, Evans-Molina C, Oram RA. Type 1 diabetes. Lancet. 2018;391(10138):2449–62. Epub 2018/06/20. doi: 10.1016/S0140-6736(18)31320-5 . 29916386 PMC6661119

[10] Lee KP, Kim YS, Yoon SA, Han K, Kim YO. Association between Blood Pressure and Renal Progression in Korean Adults with Normal Renal Function. J Korean Med Sci. 2020;35(34):e312. Epub 2020/08/31. doi: 10.3346/jkms.2020.35.e312 ; PubMed Central PMCID: PMC7458856. 32864910 PMC7458856

[11] Paul M, Poyan Mehr A, Kreutz R. Physiology of local renin-angiotensin systems. Physiol Rev. 2006;86(3):747–803. Epub 2006/07/04. doi: 10.1152/physrev.00036.2005 . 16816138

[12] Li B, Zhang Y, Wang F, Nair V, Ding F, Xiao H, et al. Urinary epidermal growth factor as a prognostic marker for the progression of Alport syndrome in children. Pediatr Nephrol. 2018;33(10):1731–9. Epub 2018/06/28. doi: 10.1007/s00467-018-3988-1 ; PubMed Central PMCID: PMC6132884. 29948307 PMC6132884

[13] Ledeganck KJ, den Brinker M, Peeters E, Verschueren A, De Winter BY, France A, et al. The next generation: Urinary epidermal growth factor is associated with an early decline in kidney function in children and adolescents with type 1 diabetes mellitus. Diabetes Res Clin Pract. 2021;178:108945. Epub 2021/07/11. doi: 10.1016/j.diabres.2021.108945 . 34245799

[14] Wong RW, Guillaud L. The role of epidermal growth factor and its receptors in mammalian CNS. Cytokine Growth Factor Rev. 2004;15(2–3):147–56. Epub 2004/04/28. doi: 10.1016/j.cytogfr.2004.01.004 . 15110798

[15] Tsau YK, Sheu JN, Chen CH, Teng RJ, Chen HC. Decreased urinary epidermal growth factor in children with acute renal failure: epidermal growth factor/creatinine ratio not a reliable parameter for urinary epidermal growth factor excretion. Pediatric research. 1996;39(1):20–4. Epub 1996/01/01. doi: 10.1203/00006450-199601000-00003 . 8825381

[16] Chou JS, Reiser IW, Porush JG. Aging and urinary excretion of epidermal growth factor. Annals of clinical and laboratory science. 1997;27(2):116–22. Epub 1997/03/01. PubMed PMID: . 9098510

[17] Zeng F, Harris RC. Epidermal growth factor, from gene organization to bedside. Semin Cell Dev Biol. 2014;28:2–11. doi: 10.1016/j.semcdb.2014.01.011 ; PubMed Central PMCID: PMC4037350. 24513230 PMC4037350

[18] Isaka Y. Epidermal growth factor as a prognostic biomarker in chronic kidney diseases. Ann Transl Med. 2016;4(Suppl 1):S62. Epub 2016/11/22. doi: 10.21037/atm.2016.10.64 ; PubMed Central PMCID: PMC5104645. 27868030 PMC5104645

[19] Yang CW, Ahn HJ, Kim WY, Shin MJ, Kim SK, Park JH, et al. Influence of the renin-angiotensin system on epidermal growth factor expression in normal and cyclosporine-treated rat kidney. Kidney Int. 2001;60(3):847–57. Epub 2001/09/05. doi: 10.1046/j.1523-1755.2001.060003847.x . 11532080

[20] Matafora V, Lanzani C, Zagato L, Manunta P, Zacchia M, Trepiccione F, et al. Urinary proteomics reveals key markers of salt sensitivity in hypertensive patients during saline infusion. J Nephrol. 2021;34(3):739–51. Epub 2021/01/06. doi: 10.1007/s40620-020-00877-z . 33398797

[21] Josefsberg Z, Ross SA, Lev-Ran A, Hwang DL. Effects of enalapril and nitrendipine on the excretion of epidermal growth factor and albumin in hypertensive NIDDM patients. Diabetes Care. 1995;18(5):690–3. Epub 1995/05/01. doi: 10.2337/diacare.18.5.690 . 8586009

[22] Shatat IF, Flynn JT. Relationships between renin, aldosterone, and 24-hour ambulatory blood pressure in obese adolescents. Pediatr Res. 2011;69(4):336–40. Epub 2010/12/24. doi: 10.1203/PDR.0b013e31820bd148 . 21178817

[23] Naets T, Vervoort L, Ysebaert M, Van Eyck A, Verhulst S, Bruyndonckx L, et al. WELCOME: improving WEight controL and CO-Morbidities in children with obesity via Executive function training: study protocol for a randomized controlled trial. BMC public health. 2018;18(1):1075. doi: 10.1186/s12889-018-5950-3 ; PubMed Central PMCID: PMC6116429. 30157826 PMC6116429

[24] Vermeiren E, Naets T, Van Eyck A, Vervoort L, Ysebaert M, Baeck N, et al. Improving Treatment Outcome in Children With Obesity by an Online Self-Control Training: A Randomized Controlled Trial. Front Pediatr. 2021;9:794256. Epub 2022/01/11. doi: 10.3389/fped.2021.794256 ; PubMed Central PMCID: PMC8733681. 35004547 PMC8733681

[25] Eyzaguirre F, Mericq V. Insulin resistance markers in children. Horm Res. 2009;71(2):65–74. Epub 2009/01/09. doi: 10.1159/000183894 . 19129710

[26] Ten S, Maclaren N. Insulin resistance syndrome in children. J Clin Endocrinol Metab. 2004;89(6):2526–39. Epub 2004/06/08. doi: 10.1210/jc.2004-0276 . 15181020

[27] Roelants M, Hauspie R, Hoppenbrouwers K. References for growth and pubertal development from birth to 21 years in Flanders, Belgium. Ann Hum Biol. 2009;36(6):680–94. Epub 2009/11/19. doi: 10.3109/03014460903049074 . 19919503

[28] Cole TJ, Bellizzi MC, Flegal KM, Dietz WH. Establishing a standard definition for child overweight and obesity worldwide: international survey. BMJ. 2000;320(7244):1240–3. Epub 2000/05/08. doi: 10.1136/bmj.320.7244.1240 ; PubMed Central PMCID: PMC27365. 10797032 PMC27365

[29] Flynn JT, Kaelber DC, Baker-Smith CM, Blowey D, Carroll AE, Daniels SR, et al. Clinical Practice Guideline for Screening and Management of High Blood Pressure in Children and Adolescents. Pediatrics. 2017;140(3). Epub 2017/08/23. doi: 10.1542/peds.2017-1904 . 28827377

[30] Zachariah JP, Graham DA, de Ferranti SD, Vasan RS, Newburger JW, Mitchell GF. Temporal trends in pulse pressure and mean arterial pressure during the rise of pediatric obesity in US children. J Am Heart Assoc. 2014;3(3):e000725. Epub 2014/05/09. doi: 10.1161/JAHA.113.000725 ; PubMed Central PMCID: PMC4309055. 24811611 PMC4309055

[31] Schwartz GJ, Munoz A, Schneider MF, Mak RH, Kaskel F, Warady BA, et al. New equations to estimate GFR in children with CKD. J Am Soc Nephrol. 2009;20(3):629–37. Epub 2009/01/23. doi: 10.1681/ASN.2008030287 ; PubMed Central PMCID: PMC2653687. 19158356 PMC2653687

[32] Meybosch S, De Monie A, Anne C, Bruyndonckx L, Jurgens A, De Winter BY, et al. Epidermal growth factor and its influencing variables in healthy children and adults. PLoS One. 2019;14(1):e0211212. Epub 2019/01/25. doi: 10.1371/journal.pone.0211212 ; PubMed Central PMCID: PMC6345470. 30677083 PMC6345470

[33] Crouch SH, Ware LJ, Gafane-Matemane LF, Kruger HS, Van Zyl T, Van der Westhuizen B, et al. Dietary sodium intake and its relationship to adiposity in young black and white adults: The African-PREDICT study. J Clin Hypertens (Greenwich). 2018;20(8):1193–202. Epub 2018/07/03. doi: 10.1111/jch.13329 ; PubMed Central PMCID: PMC8031310. 29961983 PMC8031310

[34] Jehpsson L, Sun J, Nilsson PM, Edsfeldt A, Sward P. Serum Renin Levels Increase With Age in Boys Resulting in Higher Renin Levels in Young Men Compared to Young Women, and Soluble Angiotensin-Converting Enzyme 2 Correlates With Renin and Body Mass Index. Front Physiol. 2020;11:622179. Epub 2021/02/02. doi: 10.3389/fphys.2020.622179 ; PubMed Central PMCID: PMC7844344. 33519526 PMC7844344

[35] Short KR, Blackett PR, Gardner AW, Copeland KC. Vascular health in children and adolescents: effects of obesity and diabetes. Vasc Health Risk Manag. 2009;5:973–90. Epub 2009/12/10. doi: 10.2147/vhrm.s7116 ; PubMed Central PMCID: PMC2788602. 19997578 PMC2788602

[36] Stergiou GS, Kollias A, Giovas PP, Papagiannis J, Roussias LG. Ambulatory arterial stiffness index, pulse pressure and pulse wave velocity in children and adolescents. Hypertens Res. 2010;33(12):1272–7. Epub 2010/10/01. doi: 10.1038/hr.2010.178 . 20882025

[37] Reinehr T, Kiess W, de Sousa G, Stoffel-Wagner B, Wunsch R. Intima media thickness in childhood obesity: relations to inflammatory marker, glucose metabolism, and blood pressure. Metabolism. 2006;55(1):113–8. Epub 2005/12/06. doi: 10.1016/j.metabol.2005.07.016 . 16324929

[38] Falkner B. Hypertension in children and adolescents: epidemiology and natural history. Pediatr Nephrol. 2010;25(7):1219–24. Epub 2009/05/08. doi: 10.1007/s00467-009-1200-3 ; PubMed Central PMCID: PMC2874036. 19421783 PMC2874036

[39] Li Y, Dong Y, Zou Z, Gao D, Wang X, Yang Z, et al. Association between pubertal development and elevated blood pressure in children. J Clin Hypertens (Greenwich). 2021;23(8):1498–505. Epub 2021/07/04. doi: 10.1111/jch.14315 ; PubMed Central PMCID: PMC8678653. 34216538 PMC8678653

[40] Yano Y, Sato Y, Fujimoto S, Konta T, Iseki K, Moriyama T, et al. Association of high pulse pressure with proteinuria in subjects with diabetes, prediabetes, or normal glucose tolerance in a large Japanese general population sample. Diabetes Care. 2012;35(6):1310–5. Epub 2012/04/05. doi: 10.2337/dc11-2245 ; PubMed Central PMCID: PMC3357237. 22474041 PMC3357237

[41] Dost A, Molz E, Krebs A, Bechtold S, Kapellen T, Rohrer T, et al. Pulse pressure in children and adolescents with type 1 diabetes mellitus in Germany and Austria. Pediatr Diabetes. 2014;15(3):236–43. Epub 2015/02/24. doi: 10.1111/pedi.12083 . 25705749

[42] Kwagyan J, Tabe CE, Xu S, Maqbool AR, Gordeuk VR, Randall OS. The impact of body mass index on pulse pressure in obesity. J Hypertens. 2005;23(3):619–24. Epub 2005/02/18. doi: 10.1097/01.hjh.0000160220.71350.5f . 15716705

[43] Naik AS, Le D, Aqeel J, Wang SQ, Chowdhury M, Walters LM, et al. Podocyte stress and detachment measured in urine are related to mean arterial pressure in healthy humans. Kidney Int. 2020;98(3):699–707. Epub 2020/08/03. doi: 10.1016/j.kint.2020.03.038 . 32739208 PMC10440835

[44] Lu CC, Wang GH, Lu J, Chen PP, Zhang Y, Hu ZB, et al. Role of Podocyte Injury in Glomerulosclerosis. Adv Exp Med Biol. 2019;1165:195–232. Epub 2019/08/11. doi: 10.1007/978-981-13-8871-2_10 ; PubMed Central PMCID: PMC7120923. 31399967 PMC7120923

[45] Flynn JT, Daniels SR. Pharmacologic treatment of hypertension in children and adolescents. J Pediatr. 2006;149(6):746–54. Epub 2006/12/02. doi: 10.1016/j.jpeds.2006.08.074 . 17137886

[46] Lin M, Heizhati M, Gan L, Hong J, Wu T, Xiamili Z, et al. Higher plasma renin activity is associated with increased kidney damage risk in patients with hypertension and glucose metabolic disorders. J Clin Hypertens (Greenwich). 2022;24(6):750–9. Epub 2022/05/07. doi: 10.1111/jch.14492 ; PubMed Central PMCID: PMC9180335. 35522256 PMC9180335

[47] Flannery AH, Ortiz-Soriano V, Li X, Gianella FG, Toto RD, Moe OW, et al. Serum renin and major adverse kidney events in critically ill patients: a multicenter prospective study. Crit Care. 2021;25(1):294. Epub 2021/08/16. doi: 10.1186/s13054-021-03725-z ; PubMed Central PMCID: PMC8364694. 34391450 PMC8364694

